# Intestinal crypt-derived enteroid coculture in presence of peristaltic longitudinal muscle myenteric plexus

**DOI:** 10.1093/biomethods/bpaa027

**Published:** 2020-12-23

**Authors:** Daniel E Levin, Arabinda Mandal, Mark A Fleming, Katherine H Bae, Brielle Gerry, Sean R Moore

**Affiliations:** Department of Surgery, University of Virginia, Charlottesville, VA 22908, USA; Department of Surgery, University of Virginia, Charlottesville, VA 22908, USA; Department of Surgery, University of Virginia, Charlottesville, VA 22908, USA; Department of Surgery, University of Virginia, Charlottesville, VA 22908, USA; Department of Surgery, University of Virginia, Charlottesville, VA 22908, USA; Department of Pediatrics, University of Virginia, Charlottesville, VA 22908, USA

**Keywords:** longitudinal muscle myenteric plexus (LMMP), peristalsis, enteroids, organoids, coculture, small intestine, jejunum

## Abstract

The role of enteric neurons in driving intestinal peristalsis has been known for over a century. However, in recent decades, scientists have begun to unravel additional complex interactions between this nerve plexus and other cell populations in the intestine. Investigations into these potential interactions are complicated by a paucity of tractable models of these cellular relationships. Here, we describe a novel technique for *ex vivo* coculture of enteroids, so called “mini-guts,” in juxtaposition to the longitudinal muscle myenteric plexus (LMMP). Key to this system, we developed a LMMP culture media that: (i) allows the LMMP to maintain *ex vivo* peristalsis for 2 weeks along with proliferation of neurons, glia, smooth muscle and fibroblast cells, and (ii) supports the proliferation and differentiation of the intestinal stem cells into enteroids complete with epithelial enterocytes, Paneth cells, goblet cells, and enteroendocrine cells. Importantly, this technique identifies a culture condition that supports both the metabolic needs of intestinal epithelium as well as neuronal elements, demonstrating the feasibility of maintaining these two populations in a single culture system. This sets the stage for experiments to better define the regulatory interactions of these two important intestinal cell populations.

## 1. Introduction

In 1899, Bayliss and Starling [1] described their “law of the intestine,” establishing that antegrade propulsive waves of muscular contraction, or peristalsis, occur independently of the central nervous system. In the ensuing 120+ years, the predominant function of the enteric neurons in peristalsis would be better defined, yet other potential roles for the enteric nervous system (ENS) would be largely ignored until latter parts of the 20th century. This lag is, in part, due to inherent challenges in modeling these complex interactions of the enteric neurons and intestinal epithelial cells.

Recent advances in understanding interactions between the ENS and epithelial cells have revealed important insight into ENS control of local blood flow, fluid flux, immunity and epithelial regulation [2]. Interrogating how these populations of cells interact is paramount to elucidating the nuanced physiology of this organ system and enhancing our understanding of intestinal structure and function in the pursuit of treatments for those afflicted with intestinal ailments.

To begin to understand complex interactions of the ENS with the surrounding tissues, a tractable coculture system is needed. In this manuscript, we describe a novel method for the coculture of enteric neurons from longitudinal muscle and myenteric plexus (LMMP) without disrupting glial and peristaltic smooth muscle attachments, in juxtaposition to clusters of small intestinal epithelial stems cells in crypts with supportive niche cells that self-organize into enteroids. With this improved *in vitro* model, we are now better equipped to answer questions related to complex interactions of the ENS with intestinal epithelial cells.

Traditionally, the maintenance of enteroids in culture was made possible by the addition of the growth factors EGF, Noggin, R-spondin 1, and Wnt-3a (ENRW) as originally described by Sato et al. [3] and Sate and Clevers [4]. At present, enteroids are used in laboratories throughout the world [5] to study intestinal epithelial physiology. Maintenance of myenteric neurons and smooth muscle cells in culture also requires specific factors [6–8]. The techniques described in this manuscript offer two important contributions. The first is the ability to maintain the LMMP in culture for up to 13 days with continuous peristalsing contractions. Peristalsis can be maintained with or without the coculture of enteroids. The addition of enteroids highlights the second major advantage over existing techniques in that we are able to keep these cell populations alive, in juxtaposition, creating an opportunity to study their interactions in a model unlike any other previously described.

## Materials and methods

A complete list of materials is located in Supplemental Table S1.

### Prepare media

The LMMP culture media is prepared in 50 mL aliquots using 37 mL of advanced DMEM/F12, 0.5 mL of Pen-Strep 100×, 1 mL of B27plus supplement 50×, 0.5 mL of N2 supplement 100×, 0.5 mL of HEPES 1 M, 0.5 mL Glutamax 100×, and 10 mL of FBS (20%) heat inactivated then filter sterilized using a 0.22-µm Steriflip (Supplementary Table S1).

The base LMMP Enteroid coculture media is very similar to the above and prepared in 50 mL aliquots using 42 mL of advanced DMEM/F12, 0.5 mL of Pen-Strep 100×, 1 mL of B27plus supplement 50×, 0.5 mL of N2 supplement 100×, 0.5 mL of HEPES 1 M, 0.5 mL Glutamax 100×, and 5 mL of FBS (10%) heat inactivated then filter sterilized using a 0.22-µm Steriflip.

The complete LMMP Enteroid coculture media is prepared by adding growth factor supplements (ENRW) to the base LMMP Enteroid coculture media. For every 1 mL of basal LMMP Enteroid coculture media, add 1 µL of 50 µg/mL EGF (E), 1 µL of 100 µg/mL Noggin (N), 1 µL of 1000 µg/mL R-Spondin 1 (R), and 1 µL of 100 µg/mL Wnt-3a (W) from 1,000× stocks in DPBS with 0.1% BSA (Supplementary Table S1).

### LMMP isolation

To begin, the donor mice are sacrificed in accordance with the institutional Animal Care and Use Committee. At our institution, euthanasia is performed by CO_2_ overdose (4 lbs/min, 2 min), followed by confirmatory cervical dislocation. The abdomen is cleansed with 70% ethanol then opened longitudinally. The small intestine (SI) from duodenum to ileum is avulsed from the mesentery and placed in a 10-cm petri dish containing ∼5 mL of DPBS with Pen-Strep 1× on ice. A 7-cm segment of intestine is isolated (Jejunum is depicted in this publication, though any segment of intestine could be used depending on the experimental goals) and will serve as the LMMP source. This is placed in its own DPBS dish. The lumen is cleared of fecal debris with 20 mL of ice cold DPBS/pen/strep (1×) using a 20-mL syringe with gavage needle (size 20) from proximal to distal. Once cleaned, it is transferred to clean dish with ∼5 mL of DPBS/Pen/Strep 1× on ice.

**Figure 1:**
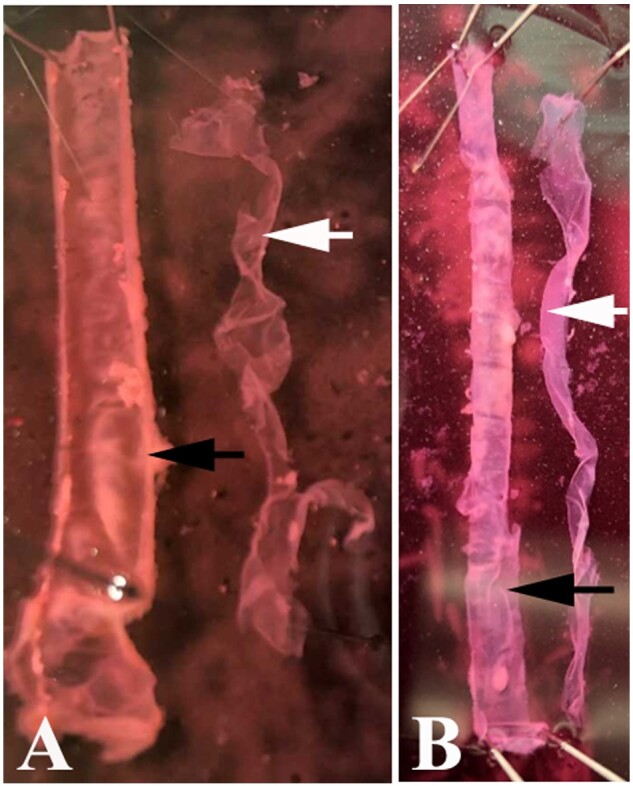
Isolation of LMMP from mouse small intestinal wall. (**A**) LMMP isolate from jejunum. (**B**) LMMP isolate from ileum. LMMP—white arrow; circular muscle and mucosa from which the LMMP was separated—black arrow.

Next, place the cleansed jejunal segment on a 9-cm silicone-coated black petri dish containing ∼30 mL of ice cold DPBS/Pen/Strep (1×). Secure the proximal and distal ends with pins and remove any remaining mesentery. With a forcep and a scissor, open the lumen along the mesenteric line and gently flatten the SI with a pair of bend forceps keeping the lumen side down. Rearrange the pins to stretch the SI as a flat sheet (see Fig. 1).

From the proximal end of the segment, create a window of LMMP near the pins by gently rubbing longitudinally along the SI outermost layer with a sterile cotton tipped applicator wet in cold DPBS. This is best performed under a dissection stereo microscope until you see the appearance of a piece of floating LMMP layer. Hold the floating LMMP layer along the entire breadth of the segment and pull the layer gently along the entire length of the SI segment to recover the entire LMMP layer from the intestinal segment using a wet cotton tipped applicator with the forceps holding the submucosal intestine down near the site of separation.

Wash the isolated LMMP in 10 mL of fresh DPBS/Pen/Strep and store in 2 mL of base coculture media on ice.

### Crypt isolation

Mince the jejunum with LMMP or without the LMMP from one or two mice into 2–3 mm pieces using scissors on a 10-cm dish with 10 mL of DPBS/Pen/Strep on ice and then transfer to a 50 mL tube on ice. Next, wash the intestinal pieces using a 10-mL pipet by pipetting in and out three times and allow the pieces to settle by gravity. Remove the supernatant by aspiration and add 10 mL of ice cold DPBS/Pen/Strep and repeat the wash procedure for ∼10 times to remove the loose villi. Remove the supernatant and add 20 mL cold crypts dissociation buffer (50 mL of this buffer is made from 49.3 mL of DPBS and 200 µL of 500 mM EDTA, and 0.5 mL Pen-Strep and stored on ice). Incubate in a cold room on a rocking platform for 25–30 min at 20 rpm. After rocking, aspirate the supernatant by gravity separation and suspend the intestinal pieces in 10 mL of ice cold crypt elution buffer (this buffer is made in 50 mL aliquots from 49.5 mL of DPBS, 50 mg BSA and 0.5 mL Pen-Strep, and stored on ice). Pass in and out of the pipet three times and allow the pieces to settle by gravity. Using a 10-mL pipet, collect the supernatant through a 70-µm strainer and mark as crypts fraction 1 and keep in ice. Repeat this step and collect crypts fractions 2–4. Next, take a 1-mL aliquot from each fraction to a 6-well plate and determine which fractions have the greatest crypts concentration using 4× objective. Centrifuge fractions 3 and 4 at 300 × *g* for 3 min at 4°C, suspend each pellet in 5 mL of base coculture media, and take 10 µL aliquots on a slide or a hemocytometer with or without a coverslip for crypt counting. The final steps are designed to create 50 µL droplets. For each of these matrigel-crypt droplets, remove ∼150 crypts (per droplet) and spin at 200 × *g* for 2 min. Next, remove the supernatant and add 10 µL of complete coculture media per droplet. Next, add 50 µL of matrigel with EGF, Noggin, R-spondin 1 (ENR), mix gently, and store on ice. As a result, drawing a 50-µL droplet from this 60 µL solution will yield approximately 125 crypts per matrigel-crypt droplet for incubation. These steps may be repeated or performed in multiples to create additional droplets as needed.

### LMMP only culture

Add 100 µL of cold matrigel in the center of a 35-mm petri dish at room temperature in a biological safety cabinet. Next, transfer a 1-cm piece of washed LMMP into the matrigel using a curved fine tip forcep and a sterile plastic loop then spread out the LMMP within the matrigel with two sterile plastic loops. Polymerize the matrigel in the 37°C CO_2_ incubator for 7 min. Immediately following, add 2 mL of warm LMMP culture media containing 20% FBS and transfer the dish in the humidified CO_2_ incubator at 37°C. Peristalsis of the LMMP can be observed within 1–2 h of incubation. Add new media every 48 h. Peristalsis of the LMMP is sensitive to temperature and better preserved when observed on a microscope equipped with a 37°C stage.

### LMMP and proliferated cells immunofluorescence

Fix the LMMP or the proliferated cells in 4% PFA in PBS at 37°C for 30 min at room temperature. The LMMPs were washed in 10 mL while LMMP-proliferated cells were washed in 5 mL of warm (37°C) DPBS three times at room temperature. The sample is then blocked and permeabilized for 60 min in 5 mL of warm (37°C) 5% normal goat serum with 0.5% Triton X-100 in DPBS (i.e., blocking buffer) at room temperature. The blocked sample is then incubated in 5.0 mL of blocking buffer containing the primary antibody or the isotype control antibody (Supplementary Table S1) at 5 µg/mL for overnight at room temperature, then washed four times in 10 mL of warm (37°C) DPBS. The probed sample is then incubated in 5.0 mL of blocking buffer containing goat anti-mouse IgG F(ab)2 Alexa Fluor 488 or Alexa Fluor 594, goat anti-rabbit IgG F(ab)2 Alexa Fluor 488, or Alexa Fluor 594 for 1 h at room temperature followed by DPBS wash. The coverslip is mounted in slow fade diamond antifade DAPI on regular or concave slides. Confocal *Z*-stack immunofluorescence images were taken in a Zeiss LSM 700 or 880 microscope.

### Propidium iodide staining of LMMP

Remove the culture media then add 2 mL of media containing 1 µg/mL of propidium iodide and incubate the LMMP sample in matrigel at 37°C for 10 min. Next, wash the sample once in 3 mL of culture media and add 2 mL of fresh media for imaging using widefield fluorescence microscope. To permeabilize a sample as a positive control, remove the media, then incubate with 2 mL of fresh media containing 2 µL of Triton X-100 and incubate at 37°C for 30 min. Remove the permeabilization solution, wash the sample with 3 mL of fresh media, and then repeat the initial steps to image.

### LMMP enteroids coculture

Add a 35-µL drop of matrigel to the center of a 35-mm glass bottom 14 mm microwell, then add a 1-cm segment of isolated LMMP. Spread it with sterile plastic loops in the biological safety cabinet, then add the 50 µL of crypts-matrigel mix on top of the LMMP culture. Next, heat the wells at 37°C for 7 min and add 2 mL of warm complete coculture media and place the dish in the humidified CO_2_ incubator at 37°C. Change the coculture media every 48 h.

### Immunofluorescence staining of murine enteroids

Fix the enteroids after removing the media in 2.5 mL of 4% PFA in PBS at 37°C for 30 min then wash the enteroids in 5 mL of DPBS, ×2 at 37°C. Next permeabilize the enteroids in 2.5 mL of DPBS with 3% BSA and 1% Triton X-100 (blocking buffer) for 1 h at room temperature. Following this, incubate the enteroids in 3 mL of primary antibody or IgG control (Supplementary Table S1) at 5 µg/mL in blocking buffer for overnight at room temperature. Wash the enteroids five times in 5 mL of warm DPBS at 37°C then incubate the enteroids in 3 mL of secondary antibody at 2 µg/mL in blocking buffer for 1 h at room temperature. Wash the enteroids five times in 5 mL of DPBS at 37°C. Finally, mount the enteroids in slow fade DAPI (∼200µL) for nuclear staining for 5 min. Gently remove the glass bottom coverslip from the 35-mm dish using a scalpel blade and place on a concavity slide and then sealed with nail polish for confocal imaging using a Zeiss LSM 700 or 880 microscope.

## Results and discussion

**Figure 2:**
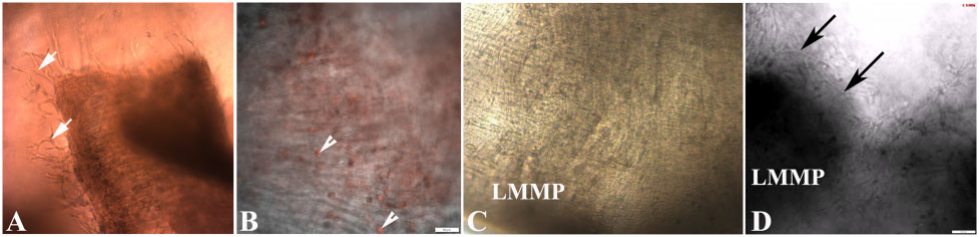
Growth of undissociated LMMP in tissue culture media. (**A**) LMMP tissue showing new cell proliferation (arrows) from the periphery following 4 days in culture (×200). (**B**) Propidium iodide staining (red, arrow heads) shows few dead cells in LMMP following 13 days in culture. (**C**) Video image of the peristaltic LMMP without proliferated cells following 2 days in culture. (**D**) Video image of the peristaltic LMMP with proliferated cells (arrows) following 13 days in culture.

**Figure 3:**
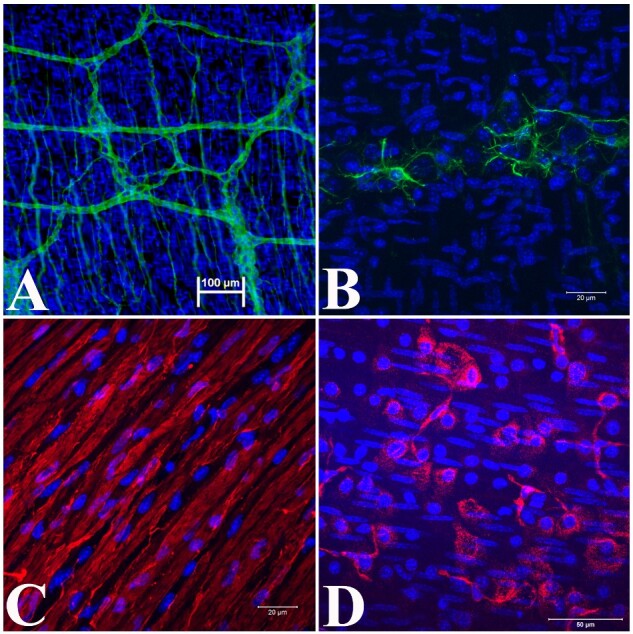
Immunofluorescence (IF) of differentiated cells in adult mouse LMMP from jejunum. (**A**) Enteric neurons forming myenteric plexus, probed with anti-Tubulin beta-3 (TUBB3, green) antibody, scale bar—100 µm. (**B**) Enteric glia cells, visualized by anti-Glial fibrillary acidic protein (GFAP, green) antibody, scale bar 20 µm. (**C**) Enteric smooth muscle cells in parallel were identified using alpha-smooth muscle actin (alpha-SMA, red) antibody, scale bar 20 µm. (**D**) Fibroblast were detected using anti-Vimentin (red) antibody, scale bar 50 µm. Each confocal IF image is a merge of DAPI (nuclear stain, blue) and Alexa Fluor 488 (A, B) or 594 (C, D).

**Figure 4:**
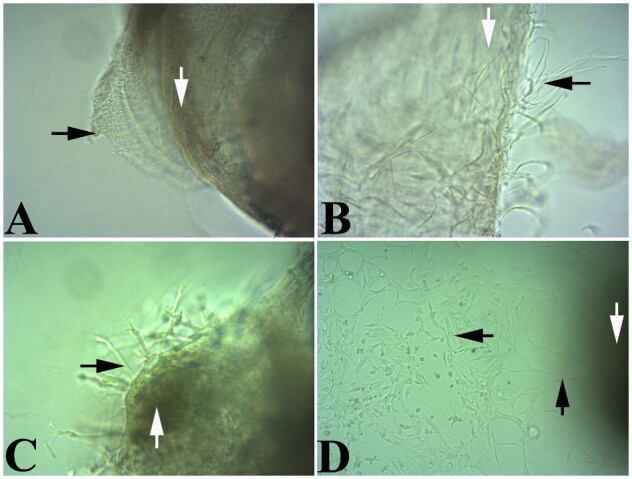
Light microscopic images of LMMP only proliferation in coculture media. (**A, B**) Early stages of new cell proliferation from LMMP in matrigel following 2 days in culture. (**C, D**) Proliferation of new cells from LMMP after 6 days in culture. Black arrows—new cell proliferation; white arrows—LMMP, partially focused; magnification—(A, B, and C) 200×; (D) 100×.

**Figure 5:**
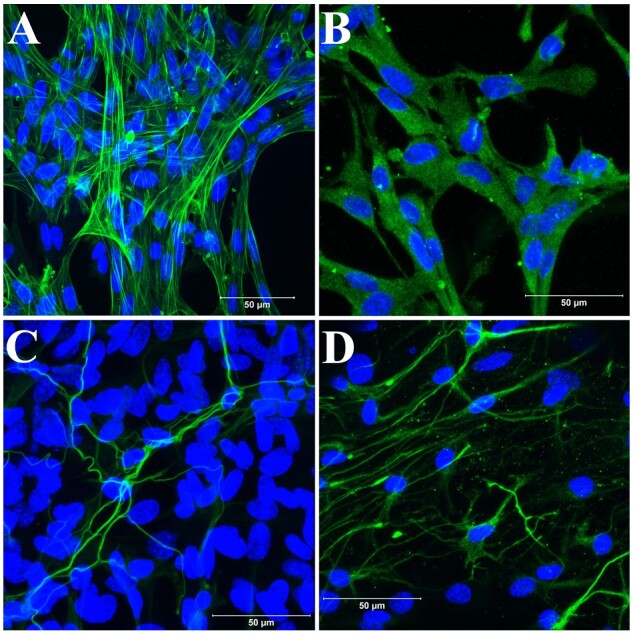
Immunofluorescent analysis of primary cells developed from LMMP only in coculture media. (**A**) Enteric smooth muscle cells were identified using anti-alpha smooth muscle actin antibody. (**B**) Enteric fibroblast were detected in the culture using anti-Vimentin antibody. (**C**) Enteric neurons were identified using anti-TUBB3 antibody. (**D**) Glia cell proliferations were confirmed using anti-GFAP antibody. The cells were fixed after 8 (A, B) or 9 (C) or 10 (D) days in culture. Each confocal image is a merge of green and DAPI nuclear stain. Scale bar 50 µm.

**Figure 6:**
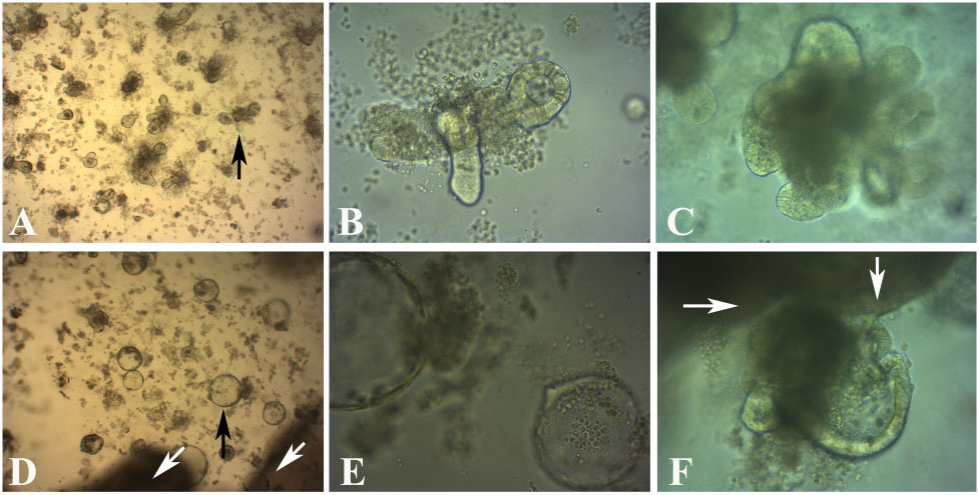
Light microscopic images of jejunum crypts coculture in presence of LMMP in matrigel produced mature enteroids. (**A**) Crypts without LMMP formed early enteroids after 2 days in culture. (**B**) High powered magnification of an early enteroid with a budding crypt from panel A (marked with a black arrow in panel A). (**C**) Crypts without LMMP formed a enteroid with budding crypts after 4 days in culture. (**D**) Crypts with LMMP formed spherical enteroids (enterospheres) near LMMP (white arrows) after 2 days in culture. (**E**) Higher magnification of spherical enteroids (enterospheres) from panel D (marked with black arrow in panel D). (**F**) Crypts with LMMP formed an enteroid with limited budding crypts after 4 days in culture. Magnification—(A and D) 40×; (B, C, E, and F) 200×.

The LMMP enteroid coculture method starts with a simple and quick isolation of LMMP from the SI (Fig. 1). The isolated LMMP reveals proliferation of new cells after 4 days (Fig. 2A) and maintains peristalsis beginning within hours and lasting up to 13 days in LMMP culture media containing 10% (Fig. 2C and Supplementary Video S1) or 20% FBS (Fig. 2D and Supplementary Video S2) without ENRW. Propidium iodide staining [9] of the 13-day-old LMMP revealed few dead cells in this media (Fig. 2B). Immunofluorescence analyses of the isolated whole mount LMMP showed the presence of neurons in the myenteric plexus, glia cells, abundant smooth muscle cells and fibroblasts (Fig. 3). The isolated LMMP supported in coculture media with 10% FBS also demonstrated peristalsis for 2–4 days and cell proliferation (Fig. 4). Phenotypic analysis of these primary cells by confocal immunofluorescence confirmed the proliferation of all major LMMP cell types including neurons, glia, fibroblast, and smooth muscle cells probed with TUBB3, GFAP, vimentin, and alpha-smooth muscle actin antibodies, respectively (Fig. 5). The enteroids grown in the coculture media without the LMMP adopt the stereotypic budding appearance (Fig. 6A–C) that has been previously described [3–5]. Interestingly, when cultured in juxtaposition with the LMMP in the coculture media, the enteroids adopt a spherical phenotype that persists for several days in culture (Fig. 6D–F). The cause of this phenotypic change is not understood and the subject of ongoing research. Interestingly, if allowed to remain in the coculture condition, the spherical enteroids will adopt the more commonly observed budded appearance after 6 days in culture (data not shown).

**Figure 7:**
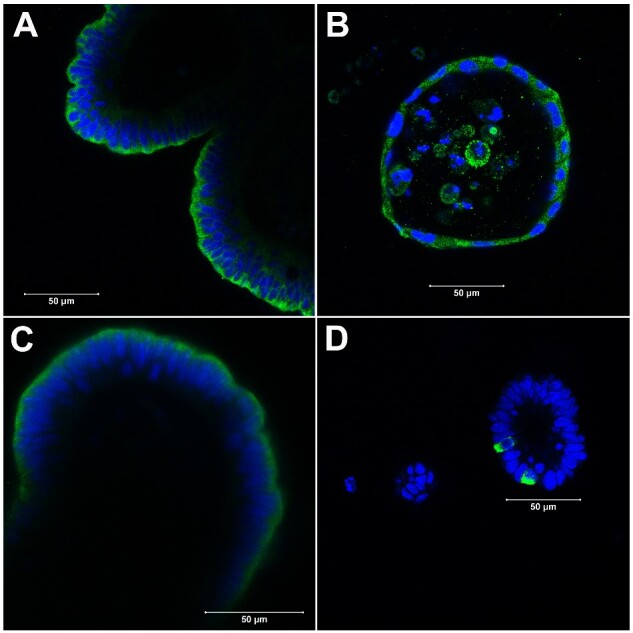
Confocal image of differentiated cell types of enteroids from LMMP coculture. (**A**) Enterocytes stained with anti-Villin antibody. (**B**) Paneth cells probed with anti-Lysozyme antibody. (**C**) Goblet cells detected with anti-Mucin 2 antibody. (**D**) Enteroendocrine cells visualized using anti-Chromogranin A antibody. Enteroids were in the LMMP coculture for day 4 (B–D) or day 6 (A). Each confocal images were merge of nuclear stain DAPI (blue) and Alexa Fluor 488 (green). Scale bar: 50 µm.

**Figure 8:**
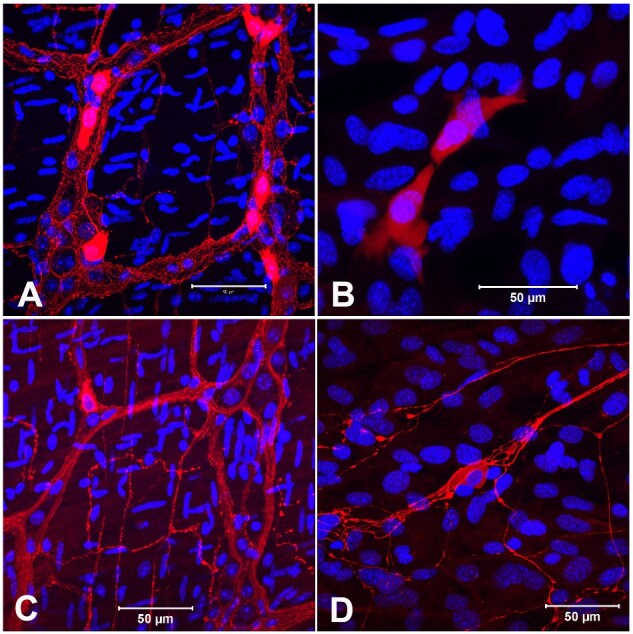
Confocal immunofluorescence images revealed enteric neurons from LMMP and LMMP proliferated cells in coculture media. The neuronal subtypes were confirmed by Calretinin (**A**, **B**) and nNOS (**C**, **D**) antibodies following 9 days (B) or 13 days (D) in culture. nNOS positive neurite networks were apparent in the culture. Each image was a merge of Alexa Fluor 594 (red) and nuclear stain DAPI (blue). Scale bar: 50 µm. A, C: from LMMP; B, D: from coculture.

All major intestinal epithelial cell types including enterocytes, Paneth cells, goblet cells, and enteroendocrine cells were found to be differentiated in the developed enteroids following coculture in presence of LMMP (Fig. 7). Control samples from normal murine SI are demonstrated in the Supplementary Material (Supplementary Fig. S1). To further characterize the neuronal subpopulation [10] in the LMMP, we stained for the presence of calcium-binding Calretinin (Fig. 8A and B) and neurotransmitter synthetic enzyme, nNOS (Fig. 8C and D) following 9 and 13 days in coculture media, respectively.

With this method, we have identified two important improvements. First, we demonstrate continued function of the neurons by persistent and sustained peristaltic contractions that we are now able to maintain for 2 weeks at a time. This offers a powerful means to study enteric neuron-mediated smooth muscle contraction. Second, we are able to describe a simple coculture technique with a single culture media capable of supporting both the neuronal and epithelial cell populations. Other authors have described methods for coculturing these populations of cells [11, 12], but they utilize transwell systems and individual cell type-specific media to initiate the coculture system. Our technique is comparatively simple, does not require a transwell culture system, and uses a single media to maintain LMMP peristalsis and the proliferation and differentiation of neuronal and epithelial cell types.

With this novel improvement, we can now combine both the neuronal elements and the epithelial cells into a unique coculture system to study their interactions. As demonstrated in the previous figures (Fig. 6D–F), we can observe the altered phenotype of those enteroids cultured in contact with the LMMP in comparison to those cultured in an identical media, but without the LMMP. The enlargement of the early enterospheres could be due to the release of some paracrine proinflammatory factors from the isolated LMMP [13]. The next stages of our work will seek to determine the cause of this phenotypic difference observed in the early enteroids and, to determine the signaling profile of a specific cell type (neuron or glia or smooth muscle cells developed at different times) with the enteroid proliferation and differentiation pattern in this coculture system.

## Data Availability

The data that support the findings of this study are available from the corresponding author, DEL, upon reasonable request.

## Supplementary data

Supplementary data are available at *Biology Methods and Protocols* online.

## Supplementary Material

bpaa027_Supplementary_Data
