# Supplementary material for: Intestinal crypt-derived enteroid coculture in presence of peristaltic longitudinal muscle myenteric plexus
Source: Biol Methods Protoc. 2020 Dec 23;6(1):bpaa027. doi: 10.1093/biomethods/bpaa027 (PMC7891127; doi:10.1093/biomethods/bpaa027)
Supplement: bpaa027_Supplementary_Data [file bpaa027_supplementary_data.zip › Supplemental Table 1.docx]

**Supplemental Table 1: Material and reagent list with preferred manufacturer and catalog number.**

| **Name** | **Company** | **Calalog Number** |
| --- | --- | --- |
| BSA | Millipore Sigma | A7030 |
| DPBS | ThermoFisher Scientific | 14190144 |
| EDTA | ThermoFisher Scientific | 15575038 |
| HBSS | ThermoFisher Scientific | 14170-112 |
| Krebs-Ringer Solution | Fisher Scientific | AAJ67591AP |
| Advanced DMEM/F12 | ThermoFisher Scientific | 12634-010 |
| Pen-Strep | ThermoFisher Scientific | 15140122 |
| B27 plus supplement | ThermoFisher Scientific | A3582801 |
| N2 supplement | ThermoFisher Scientific | 17502048 |
| HEPES | ThermoFisher Scientific | 15630080 |
| Glutamax | ThermoFisher Scientific | 35050061 |
| FBS heat inactivated | ThermoFisher Scientific | 10082139 |
| Steriflip-GP sterile centrifuge tube top filter | Millipore Sigma | SCGP00525 |
| Sterile polystyrene loops | Fisher Scientific | 22170-201 |
| Sterile cotton tipped applicators | Fisher Scientific | 22029-504 |
| Silicone coated black petri dish | Living Systems Instrumentation | DD-90-S-BLK; DD-50-S-BLK |
| Reversible strainer 70 µm | Stemcell Technologies | 27260 |
| Matrigel GFR | Fisher Scientific | CB-40230C |
| EGF protein CF | ThermoFisher Scientific | PMG8041 |
| Noggin protein CF | R&D systems | 1967-NG-025 |
| R-Spondin 1 protein CF | R&D systems | 3474-RS-050 |
| Wnt-3a protein CF | R&D systems | 1324-WN-010/CF |
| Petri dishes 35 mm with glass bottom | MatTek | P35G-1.5-14-C |
| Concavity slides | Fisher Scientific | 50-949-425 |
| PFA, 4% in PBS | Fisher Scientific | NC1266351 |
| Hydrophobic barrier PAP pen | Millipore Sigma | Z377821 |
| Triton X-100 | Millipore Sigma | T8787 |
| Propidium iodide | ThermoFisher Scientific | P3566 |
| Normal goat serum | ThermoFisher Scientific | 10000C |
| Slow Fade DAPI | ThermoFisher Scientific | S36964 |
| TUBB3, mouse monoclonal antibody | BioLegend | 801202 |
| GFAP, mouse monoclonal antibody | Millipore Sigma | MAB3402 |
| Alpha smooth muscle actin, mouse monoclonal antibody | SigmaAldrich | A5228 |
| Vimentin, mouse monoclonal antibody | Abcam | ab8978 |
| Villin, rabbit recombinant monoclonal antibody | Abcam | ab130751 |
| Lysozyme, rabbit recombinant monoclonal antibody | ThermoFisher Scientific | MA5-32154 |
| Mucin 2, mouse monoclonal antibody | ThermoFisher Scientific | MA5-12345 |
| Chromogranin A, rabbit polyclonal antibody | Abcam | ab45179 |
| Calretinin, rabbit monoclonal antibody | ThermoFisher Scientific | MA5-14540 |
| nNOS, rabbit polyclonal antibody | Enzo | ALX-210-529 |
| Mouse IgG1 | BioLegend | 401402 |
| Mouse IgG2a | BioLegend | 401502 |
| Rabbit IgG | ThermoFisher Scientific | 02-6102 |
| Goat anti-mouse IgG F(ab)2 Alexa Fluor 488 | Jackson ImmunoResearch | 115-546-062 |
| Goat anti-mouse IgG F(ab)2 Alexa Fluor 594 | Jackson ImmunoResearch | 115-586-062 |
| Goat anti-rabbit IgG F(ab)2 Alexa Fluor 488 | Jackson ImmunoResearch | 111-546-144 |
| Goat anti-rabbit IgG F(ab)2 Alexa Fluor 594 | Jackson ImmunoResearch | 111-586-144 |
