# Supplementary material for: Intestinal crypt-derived enteroid coculture in presence of peristaltic longitudinal muscle myenteric plexus
Source: Biol Methods Protoc. 2020 Dec 23;6(1):bpaa027. doi: 10.1093/biomethods/bpaa027 (PMC7891127; doi:10.1093/biomethods/bpaa027)
Supplement: bpaa027_Supplementary_Data [file bpaa027_supplementary_data.zip › Levin supplementary figure 1.pdf]

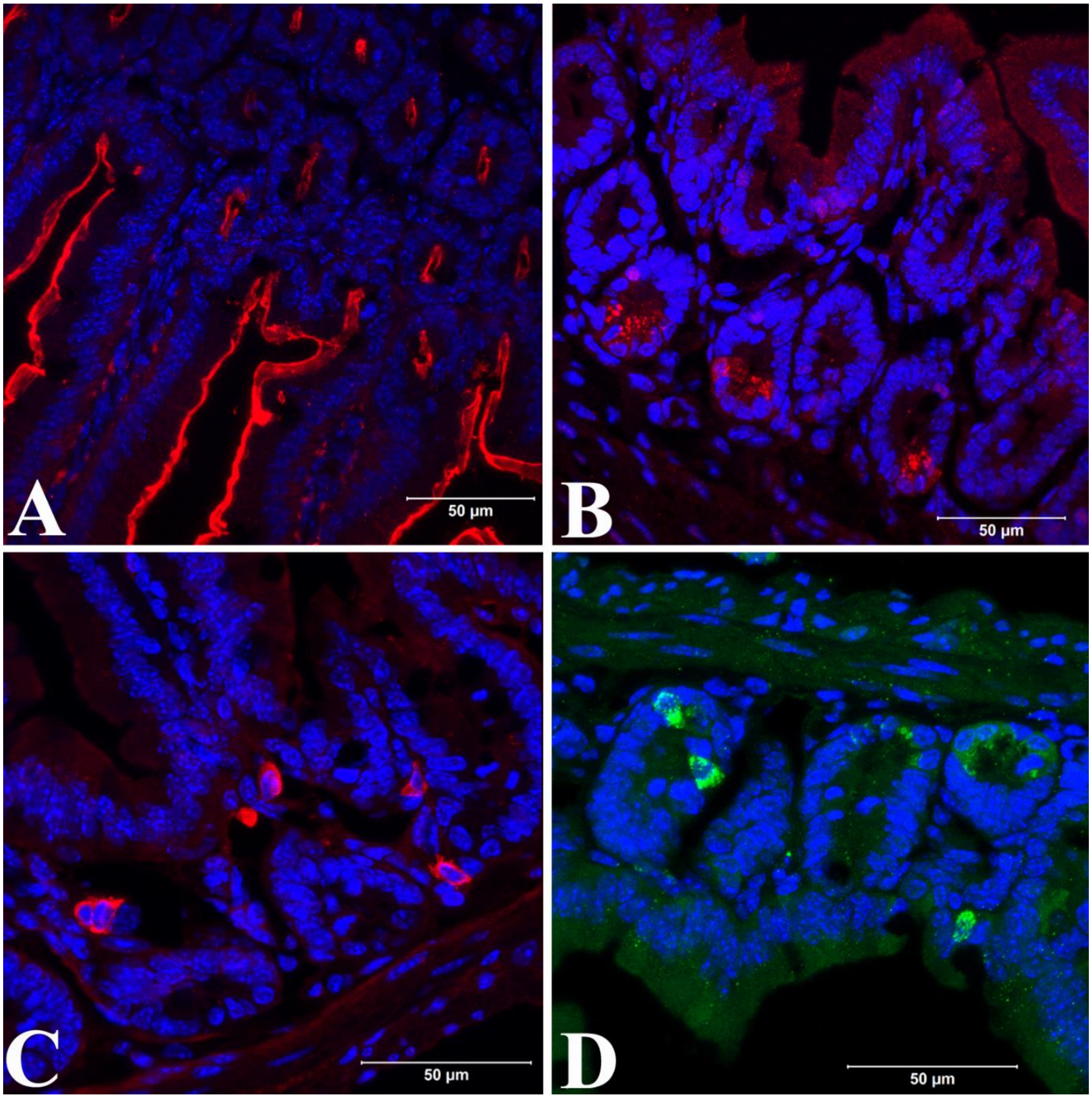

Supplemental Fig. 1: Immunofluorescent staining of mouse intestinal epithelial cells. **(A):** Enterocytes lining the villi and in crypts (red) were detected using anti-Villin antibody; **(B):** Paneth cells at the bottom of the crypts were detected using anti-Lysozyme antibody (red); **(C):** Goblet cells secreting mucin in villi and crypts were detected using anti-Mucin 2 antibody (red); **(D):** Enteroendocrine cells secretes hormones were detected in crypts and villi using anti-Chromogranin A antibody (green). Each confocal images were merge of DAPI (nuclear stain, blue) and Alexa Fluor 488 (D) or 594 (A, B, C). Scale bar: 50 µm.
