# Supplementary material for: Intestinal crypt-derived enteroid coculture in presence of peristaltic longitudinal muscle myenteric plexus
Source: Biol Methods Protoc. 2020 Dec 23;6(1):bpaa027. doi: 10.1093/biomethods/bpaa027 (PMC7891127; doi:10.1093/biomethods/bpaa027)
Supplement: bpaa027_Supplementary_Data [file bpaa027_supplementary_data.zip › Supplemental Videos.docx]

**Supplemental Video. 1:** LMMP (without newly proliferated cells) peristalsis video after 2 days in LMMP culture media with 10% FBS.

**Supplemental Video. 2:** LMMP (with newly proliferated cells) peristalsis video after 13 days in LMMP culture media with 20% FBS.
